# VEGF, but Not BDNF, Prevents the Downregulation of KCC2 Induced by Axotomy in Extraocular Motoneurons

**DOI:** 10.3390/ijms25189942

**Published:** 2024-09-14

**Authors:** Jaime Capilla-López, Rosendo G. Hernández, Génova Carrero-Rojas, Paula M. Calvo, Francisco J. Alvarez, Rosa R. de la Cruz, Angel M. Pastor

**Affiliations:** 1 Departamento de Fisiología, Facultad de Biología, Universidad de Sevilla, 41012 Sevilla, Spain; jcapilla@us.es (J.C.-L.); rgarcia20@us.es (R.G.H.); paulamcalvo@us.es (P.M.C.); rmrcruz@us.es (R.R.d.l.C.); 2 Center for Anatomy and Cell Biology, Division of Anatomy, Medical University Vienna, 1090 Vienna, Austria; genova.carrerorojas@meduniwien.ac.at; 3 Department of Cell Biology, Emory University, Atlanta, GA 30322, USA; francisco.j.alvarez@emory.edu

**Keywords:** oculomotor system, cation–chloride cotransporters, nerve injury, choline acetyltransferase (ChAT), vascular endothelial growth factor, brain-derived neurotrophic factor, GABA depolarization, neurological diseases, chloride homeostasis, NKCC1

## Abstract

The potassium–chloride cotransporter KCC2 is the main extruder of Cl^-^ in neurons. It plays a fundamental role in the activity of the inhibitory neurotransmitters (GABA and glycine) since low levels of KCC2 promote intracellular Cl^-^ accumulation, leading to the depolarizing activity of GABA and glycine. The downregulation of this cotransporter occurs in neurological disorders characterized by hyperexcitability, such as epilepsy, neuropathic pain, and spasticity. KCC2 is also downregulated after axotomy. If muscle reinnervation is allowed, the KCC2 levels recover in motoneurons. Therefore, we argued that target-derived neurotrophic factors might be involved in the regulation of KCC2 expression. For this purpose, we performed the axotomy of extraocular motoneurons via the monocular enucleation of adult rats, and a pellet containing either VEGF or BDNF was chronically implanted in the orbit. Double confocal immunofluorescence of choline acetyl-transferase (ChAT) and KCC2 was carried out in the brainstem sections. Axotomy led to a KCC2 decrease in the neuropil and somata of extraocular motoneurons, peaking at 15 days post-lesion, with the exception of the abducens motoneuron somata. VEGF administration prevented the axotomy-induced KCC2 downregulation. By contrast, BDNF either maintained or reduced the KCC2 levels following axotomy, suggesting that BDNF is involved in the axotomy-induced KCC2 downregulation in extraocular motoneurons. The finding that VEGF prevents KCC2 decrease opens up new possibilities for the treatment of neurological disorders coursing with neuronal hyperactivity due to KCC2 downregulation.

## 1. Introduction

The potassium–chloride cotransporter 2 (KCC2) is the major chloride extruder in neurons, being responsible for the low intracellular concentration of this anion necessary for GABA and glycine hyperpolarizing actions [1,2,3,4]. This function is opposed by sodium–potassium–chloride cotransporter 1 (NKCC1), and in immature neurons, the predominance of NKCC1 activity over that of KCC2 leads to a high intracellular chloride concentration, which generates the driving force for this anion to exit the cell, causing GABA and glycine membrane depolarizations [5,6,7]. During development, the GABA and glycine actions switch to hyperpolarization because of the upregulation of the KCC2 expression [6,8,9,10] or the functional downregulation of NKCC1, as occurs in motoneurons [11,12]. The KCC2 levels and activity in adult neurons are not constant and can decrease in many neurological disorders causing hyperexcitability states [13,14,15,16,17,18]. KCC2 decrease is also associated with psychiatric diseases such as schizophrenia and autism spectrum disorders [16,19,20]. In the case of motoneurons, the partial removal of the KCC2 membrane content after spinal cord injury results in disinhibition and spasticity [21,22,23].

Motoneurons axotomized after peripheral nerve injury are an extreme case because they stop expressing *kcc2* mRNA, resulting in the profound removal of KCC2 in the spinal [24,25,26,27] and brainstem motoneurons of the facial [28,29], hypoglossal [30], and dorsal vagal motor nuclei [31]. This causes spontaneous GABA depolarizations in motoneurons disconnected from muscle [28,30]. Recently, we extended these findings to the oculomotor system, showing that injured oculomotor and trochlear motoneurons decrease the KCC2 content on their somatic membranes [32].

The motor axon reinnervation of muscle leads to KCC2 recovery in motoneurons [25,26,29,30], suggesting that muscle-derived neurotrophic factors could play important roles regulating KCC2 levels. Two potential muscle-derived neurotrophic factors are brain-derived neurotrophic factor (BDNF) and vascular endothelial growth factor (VEGF), and both have been implicated in KCC2 regulation. The actions of BDNF on KCC2 are diverse and conflicting. The BDNF action on KCC2 is context- and neuron-dependent: BDNF upregulates KCC2 during postnatal development [10,33,34], but in many adult neurons, BDNF downregulates or upregulates KCC2 depending on whether they are intact or injured, respectively [21,35,36,37,38]. However, other authors have described that BDNF downregulates KCC2 after injury [39,40,41,42] and mediates the decrease in KCC2 observed in neurological diseases, such as epilepsy, neuropathic pain, and spasticity [15,43,44,45]. Blocking endogenous BDNF-TrkB signaling in spinal motoneurons, either pharmacologically or genetically, does not prevent KCC2 downregulation after axotomy [25], but whether exogenous BDNF modifies KCC2 expression is unknown. Fewer studies have analyzed the role of VEGF. We recently reported that VEGF administration increases KCC2 in axotomized abducens motoneurons, and this correlates with increased inhibitory discharge modulation in these motoneurons. Surprisingly, abducens motoneurons are exceptional in that they do not downregulate KCC2 after axotomy [32].

The goal of the present work was to investigate the influence of VEGF and BDNF, exogenously applied, on KCC2 regulation in all three oculomotor nuclei after peripheral nerve injury. Our findings show that whereas VEGF prevented the decrease in KCC2 after axotomy, BDNF either did not affect or enhanced the KCC2 downregulation after injury.

## 2. Results

### 2.1. Time Course of Changes in KCC2 Levels after Axotomy of Extraocular Motoneurons

**Figure 1:**
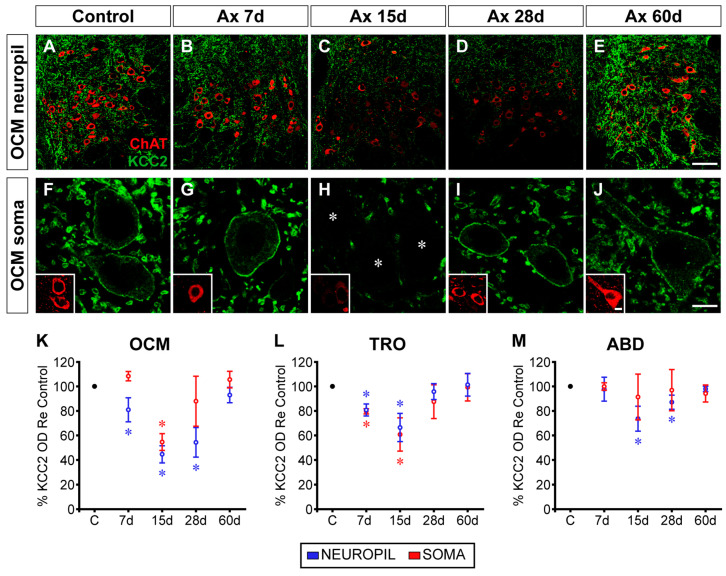
Time course of changes in KCC2 levels after axotomy of extraocular motoneurons. (**A**–**E**) Low-magnification confocal images of the oculomotor nucleus (OCM) in control (**A**) and at 7 days (7d) (**B**), 15 days (**C**), 28 days (**D**), and 60 days (**E**) after axotomy (Ax). (**F**–**J**) Same as (**A**–**E**) but for images at higher magnification to illustrate KCC2 around the soma surface of motoneurons. For (**A**–**J**)*,* double immunofluorescence for ChAT (red) and KCC2 (green) is shown. Asterisks in (**H**) point to three motoneurons whose KCC2 staining was extremely weak. (**K**–**M**) Quantification of KCC2 optical densities (ODs) in relation (Re) to control (in percentages) at different time intervals after axotomy in the three extraocular motor nuclei: oculomotor (**K**), trochlear (TRO, (**L**)), and abducens (ABD, (**M**)). Measurements in the neuropil and in the soma plasma membrane are illustrated in blue and red, respectively. * indicates significant difference (*p* < 0.05) with respect to the control (**C**) (one-way ANOVA test, followed by Fisher´s post hoc test). Data represent mean ± SD. The number of animals per group was *n* = 3. Scale bars are 75 µm in (**E**) for (**A**–**E**), 5 µm in (**J**) for (**F**–**J**), and 5 µm in insert (**J**) for all inserts.

A time course of the KCC2 expression in ChAT-identified motoneurons was performed for each nucleus studied (Figure 1). Figure 1A–E illustrate low-magnification confocal images at 7, 15, 28, and 60 days post-lesion of the oculomotor nucleus in the control (Figure 1A) and on the operated side (Figure 1B–E). A weak reduction in KCC2 immunolabeling could be appreciated at 7 days after injury (Figure 1B). The decrease in KCC2 labeling was much more conspicuous at 15 and 28 days (Figure 1C,D). Interestingly, 60 days after injury, the immunofluorescence appeared similar to that of the control (Figure 1E), thereby indicating a recovery in the KCC2 levels 2 months post-injury. To measure the KCC2 labeling on the soma surface of motoneurons, we captured images at higher magnification, as shown in Figure 1F–J for motoneurons of the oculomotor nucleus at the same post-lesion time intervals. Oculomotor motoneurons appeared with very weak immunostaining in their somatic membrane mainly at 15 days post-axotomy (Figure 1H). KCC2 labeling recovered at longer time intervals (Figure 1I,J).

We measured the KCC2 optical densities in the neuropil and the somatic plasma membrane of the three nuclei and compared them with respect to the control by using a one-way ANOVA test followed by Fisher’s post hoc test, which revealed the following significant differences (indicated by asterisks in Figure 1K–M): OCM neuropil (*F*_(4,10)_ = 26.620; *p* < 0.0001; Figure 1K); OCM somata (*F*_(4,10)_ = 13.870; *p* = 0.0004; Figure 1K); TRO neuropil (*F*_(4,10)_ = 11.810; *p* = 0.0008; Figure 1L); TRO somata (*F*_(4,10)_ = 7.876; *p* = 0.0039; Figure 1L); and ABD neuropil (*F*_(4,10)_ = 12.100; *p* = 0.0008; Figure 1M). KCC2 downregulation peaked at 15 days post-axotomy. At this time, the mean KCC2 optical density on the soma surface decreased to 54.59 ± 6.79% (± SD), and it decreased to 60.72 ± 13.55% in the oculomotor and trochlear motoneurons, respectively. In the neuropil, the optical densities dropped significantly to 44.58 ± 6.93%, 66.42 ± 11.51%, and 73.63 ± 10.10% in the oculomotor, trochlear, and abducens nuclei, respectively, at 15 days post-surgery.

The axotomized abducens motoneurons did not show significant differences in the KCC2 levels in their somatic membrane compared to the control (*p* = 0.8677; Figure 1M). This result is consistent with our previous findings in cats and rats [32]. The peculiar response of axotomized abducens motoneurons contrasts markedly with other injured motoneuron pools, such as spinal motoneurons [25], as well as other cranial motoneurons (facial, hypoglossal, and dorsal vagal motor nuclei) [28,29,30,31], including the rest of the extraocular motoneurons (oculomotor, trochlear) [32] (present results). Overall (except for abducens motoneuron cell bodies), the present results indicated that nerve injury induced a downregulation in KCC2, in both the dendritic (neuropil) and somatic compartments of motoneurons, with the maximum downregulation occurring at 15 days post-lesion, and subsequently, KCC2 began to recover until full restoration by 60 days. The fall in KCC2 was deeper in the oculomotor nucleus, intermediate in the trochlear nucleus, and the least severe in the abducens nucleus.

### 2.2. Neuropil Changes in KCC2 7 Days after Axotomy Plus VEGF or BDNF Administration

**Figure 2:**
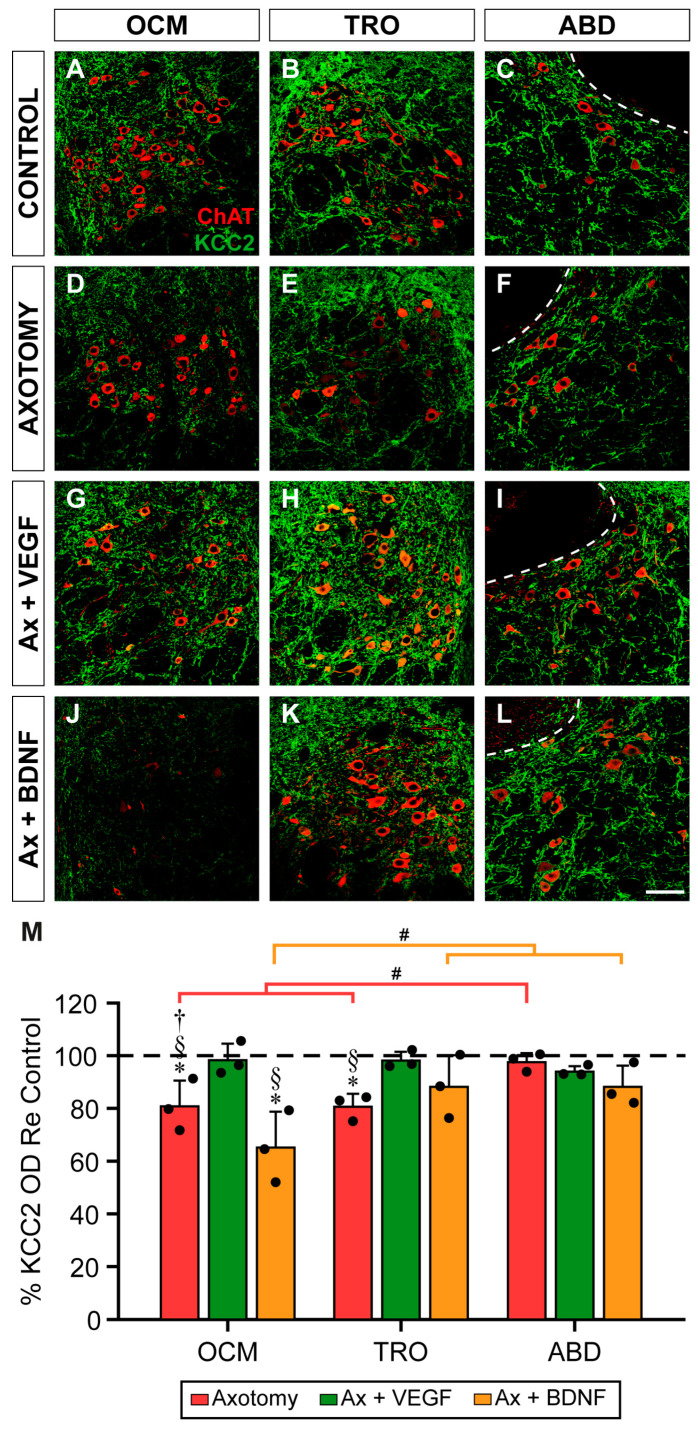
KCC2 immunofluorescence in the neuropil of extraocular motor nuclei 7 days after axotomy and axotomy with VEGF or BDNF treatment. Confocal images showing KCC2 immunofluorescence (green) in the oculomotor (OCM; (**A**,**D**,**G**,**J**)), trochlear (TRO; (**B**,**E**,**H**,**K**)), and abducens (ABD; (**C**,**F**,**I**,**L**)) nuclei. Extraocular motoneurons were identified by ChAT (red). KCC2 immunofluorescence is shown for control (**A**–**C**), 7 days after axotomy (**D**–**F**), and 7 days after axotomy (Ax) + VEGF (**G**–**I**) or axotomy + BDNF (**J**–**L**). The dashed white lines in (**C**,**F**,**I**,**L**) delimit the genu of the facial nerve. (**M**) Bar chart illustrating KCC2 optical densities (ODs) in the neuropil of the oculomotor, trochlear, and abducens nuclei 7 days after axotomy and axotomy with VEGF or BDNF delivery. Data are expressed as percentages relative (Re) to the control side (100%, dashed horizontal line). The following symbols were used to indicate significant differences between treatments within the same motor nucleus: *, significant difference (*p* < 0.05) compared to control; §, significant difference (*p* < 0.05) with respect to axotomy + VEGF; †, significant difference (*p* < 0.05) with respect to the axotomy + BDNF group. Hashtags were used to indicate significant differences (#, *p* < 0.05) between the three extraocular motor nuclei within the same treatment (two-way ANOVA test followed by Fisher´s post hoc test for multiple comparisons). Data correspond to mean ± SD. The number of animals per group was *n* = 3 (black dots). Scale bar is 75 µm in (**L**) for (**A**–**L**).

Seven days post-lesion, the faint staining of KCC2 could be appreciated in the neuropil of the oculomotor and trochlear nuclei (Figure 2D,E, respectively) compared to the control (Figure 2A,B). The administration of VEGF at the time of axotomy prevented this decrease, as observed in Figure 2G for the oculomotor nucleus and in Figure 2H for the trochlear nucleus. By contrast, BDNF treatment reduced the KCC2 immunofluorescence in the injured oculomotor nucleus (Figure 2J) as compared to the control (Figure 2A) and axotomy states (Figure 2D). In the trochlear nucleus, however, BDNF treatment did not produce any decrease in the KCC2 labeling (Figure 2K) compared to the control (Figure 2B) and axotomy states (Figure 2E). Strikingly, the abducens nucleus showed no obvious change in the intensity of immunolabeling between the different situations: control (Figure 2C), axotomy (Figure 2F), axotomy + VEGF (Figure 2I), and axotomy + BDNF (Figure 2L).

To compare the different groups, we performed a two-way ANOVA test (factors: motor nuclei and treatments). The ANOVA test was followed by Fisher´s post hoc test for multiple pairwise comparisons. There were significant differences between the nuclei (*F*_(2,24)_ = 5.001; *p* = 0.0153) and between the treatments (*F*_(3,24)_ = 15.125; *p* < 0.0001). Results are represented in Figure 2M. Concerning the neuropil measurements at 7 days post-axotomy, the data showed a significant decay in KCC2 in the oculomotor (*p* = 0.0026; 80.89 ± 9.83%) and trochlear (*p* = 0.0024; 80.74 ± 4.92%) nuclei as compared to the control (horizontal dashed line in Figure 2M, 100%). However, there was no change in the KCC2 optical density in the neuropil of the abducens nucleus 7 days after injury (*p* = 0.689; 97.70 ± 3.43%).

VEGF treatment significantly prevented the injury-induced decrease in KCC2 that occurred in the neuropil of the oculomotor and trochlear nuclei by 7 days (*p* = 0.0051) in both cases compared to axotomy (Figure 2M), presenting values similar to those of the control. In marked contrast, BDNF had no effect on the trochlear nucleus versus the control (*p* = 0.195; Figure 2M), but it further reduced the KCC2 levels by an additional 19.33% in the oculomotor nucleus compared to the axotomy state (*p* = 0.0112; Figure 2M). Finally, the abducens nucleus was the exception, as axotomy did not downregulate the KCC2 levels in the neuropil 7 days after injury, and neither VEGF nor BDNF administration altered the KCC2 values compared to the control and axotomy.

Comparisons between nuclei within the same treatment yielded two significant differences. First, at 7 days post-axotomy, the KCC2 optical density in the neuropil of the abducens nucleus was similar to that of the control and statistically higher than that in the oculomotor (*p* = 0.0069) and trochlear (*p* = 0.0065) nuclei (Figure 2M). Second, in the axotomy + BDNF situation, the oculomotor nucleus presented a significant decrease compared to the trochlear (*p* = 0.0005) and abducens (*p* = 0.0005) nuclei, and, in turn, these two latter nuclei did not differ from the control (Figure 2M).

### 2.3. Effects of Axotomy and VEGF or BDNF Administration in KCC2 Levels on the Soma Surface of the Motoneurons at 7 Days Post-Lesion

**Figure 3:**
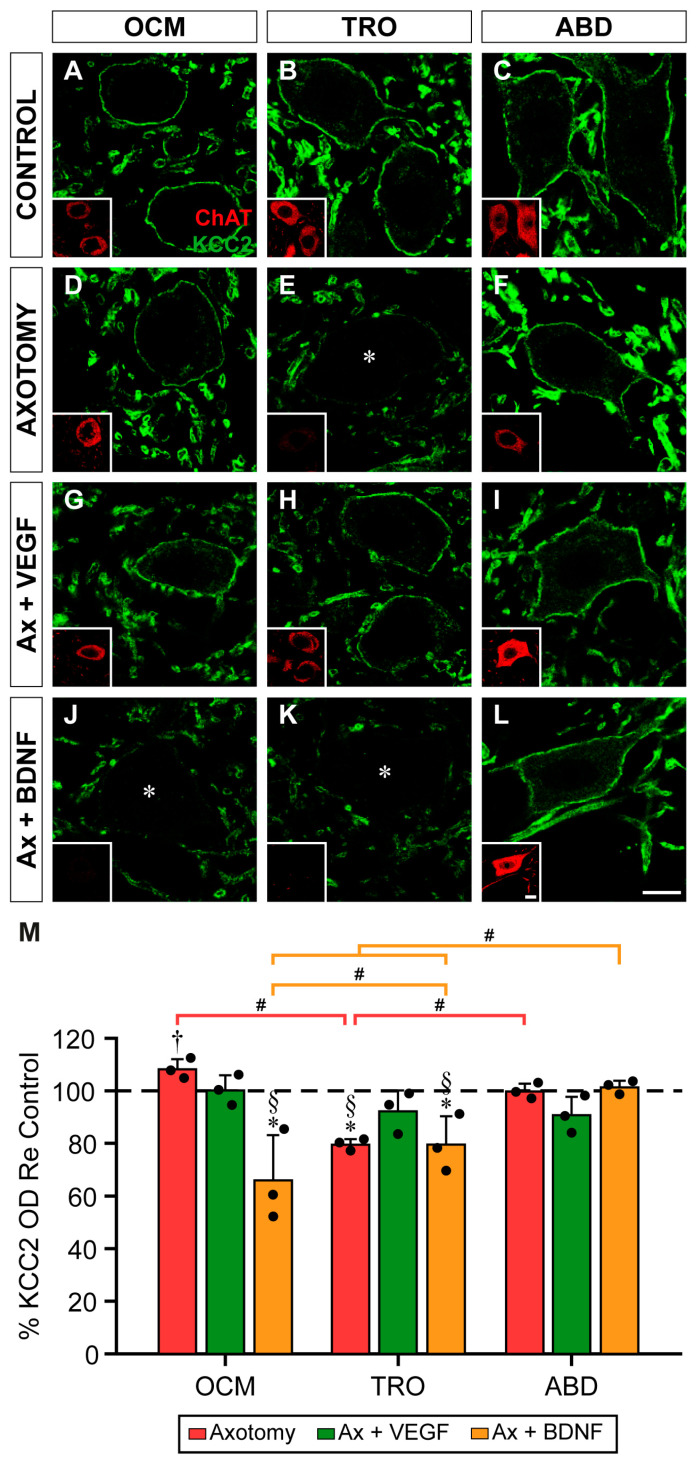
KCC2 immunofluorescence in the somatic membrane of extraocular motoneurons 7 days after axotomy and axotomy plus VEGF or BDNF. (**A**–**L**) Confocal images of oculomotor (OCM; (**A**,**D**,**G**,**J**)), trochlear (TRO; (**B**,**E**,**H**,**K**)), and abducens (ABD; (**C**,**F**,**I**,**L**)) motoneurons after immunostaining against KCC2 (green), in control (**A**–**C**), 7 days after axotomy (**D**–**F**), and 7 days after axotomy (Ax) + VEGF (**G**–**I**) or axotomy + BDNF (**J**–**L**). Inserts in each panel illustrate the same motoneuron/s identified by ChAT (red) immunoreactivity. Asterisks in (**E**,**J**,**K**) point to motoneurons whose KCC2 perisomatic staining was very faint. (**M**) Bar chart showing KCC2 optical density (OD) on the soma surface of extraocular motoneurons normalized with respect to (Re) the control side (100%, dashed horizontal line) and expressed as percentages in the following situations: axotomy, axotomy + VEGF, or axotomy + BDNF. To illustrate significant differences between treatments within the same motor nucleus, the following symbols were used: *, significant difference (*p* < 0.05) versus control; §, significant difference (*p* < 0.05) versus axotomy + VEGF; †, significant difference (*p* < 0.05) versus axotomy + BDNF. Hashtags were used to mark significant differences (#, *p* < 0.05) between the three types of extraocular motoneurons within the same treatment (two-way ANOVA test followed by Fisher´s post hoc test for multiple comparisons). Data correspond to mean ± SD. The number of animals per group was *n* = 3 (black dots). Scale bars are 5 µm in (**L**) for (**A**–**L**) panels, and 5 µm in the insert in (**L**) for all inserts.

KCC2 immunofluorescence in the control motoneurons completely outlined their perimeters (Figure 3A–C). Axotomy reduced the intensity of the perisomatic KCC2 immunofluorescence in the trochlear motoneurons (Figure 3E), while the axotomized oculomotor and abducens motoneurons did not appear to reduce their labeling (Figure 3D,F, respectively). Exogenous VEGF application (Figure 3G–I) maintained the KCC2 levels in the trochlear motoneurons similar to the control values (Figure 3H). However, images obtained in the axotomy + BDNF situation revealed the weak staining of KCC2 delineating the plasma membrane of the oculomotor (Figure 3J) and trochlear (Figure 3K) motoneurons. By contrast, the abducens motoneurons (Figure 3L) showed a similar labeling to that of the control (Figure 3C).

A two-way ANOVA test followed by Fisher´s post hoc test for multiple comparisons showed significant differences within the experimental situations (*F*_(3,24)_ = 10.388; *p* = 0.0001) and nuclei (*F*_(2,24)_ = 6.246; *p* = 0.0065).

At this time point, axotomy was not followed by a reduction in the perisomatic KCC2 optical density in comparison to the control in the oculomotor motoneurons (Figure 3M; *p* = 0.1607). The administration of VEGF also rendered values similar to those of the control (*p* = 0.9562). Strikingly, axotomy plus BDNF treatment did reduce the KCC2 optical density in the oculomotor motoneurons to 66.1 ± 17.25% as compared to the control (*p* < 0.0001; Figure 3M).

In the trochlear motoneurons, the perisomatic KCC2 optical density decreased significantly (*p* = 0.0017) to 79.62 ± 2.14% (Figure 3M) at 7 days post-lesion compared to the control. Interestingly, the VEGF supply to the axotomized trochlear motoneurons significantly (*p* = 0.0365) prevented this downregulation, yielding a result of 92.37 ± 7.95%, which was similar to that of the control (*p* = 0.1973). By contrast, BDNF treatment to the axotomized trochlear motoneurons produced a value (79.68 ± 10.84%) that was similar to that of axotomy alone (*p* = 0.9919) (Figure 3M). In the abducens motoneurons 7 days after injury, the perisomatic KCC2 optical density was similar (*p* > 0.05) between the three experimental conditions (axotomy, axotomy + VEGF, and axotomy + BDNF) as well as to that of the control (Figure 3M).

Comparison between nuclei within the same treatment revealed that the trochlear motoneurons were the only ones that downregulated their perisomatic KCC2 values 7 days after axotomy in contrast to the oculomotor (*p* < 0.0001) and abducens (*p* = 0.0017) motoneurons (Figure 3M). In the three motoneuronal types, the axotomy + VEGF situation yielded similar responses; that is, their KCC2 data were similar (*p* > 0.05) to their respective controls (Figure 3M). Regarding the axotomy + BDNF situation, the oculomotor and trochlear motoneurons showed significantly lower perisomatic KCC2 values than those of the abducens motoneurons (*p* < 0.0001 and *p* = 0.0009, respectively), which showed no change compared to the control. In turn, the oculomotor motoneurons showed a KCC2 downregulation that was significantly greater than that of the trochlear motoneurons (*p* = 0.0265).

### 2.4. Changes in KCC2 Levels after Axotomy and VEGF or BDNF Administration in the Neuropil of Extraocular Motor Nuclei at 15 Days Post-Lesion

**Figure 4:**
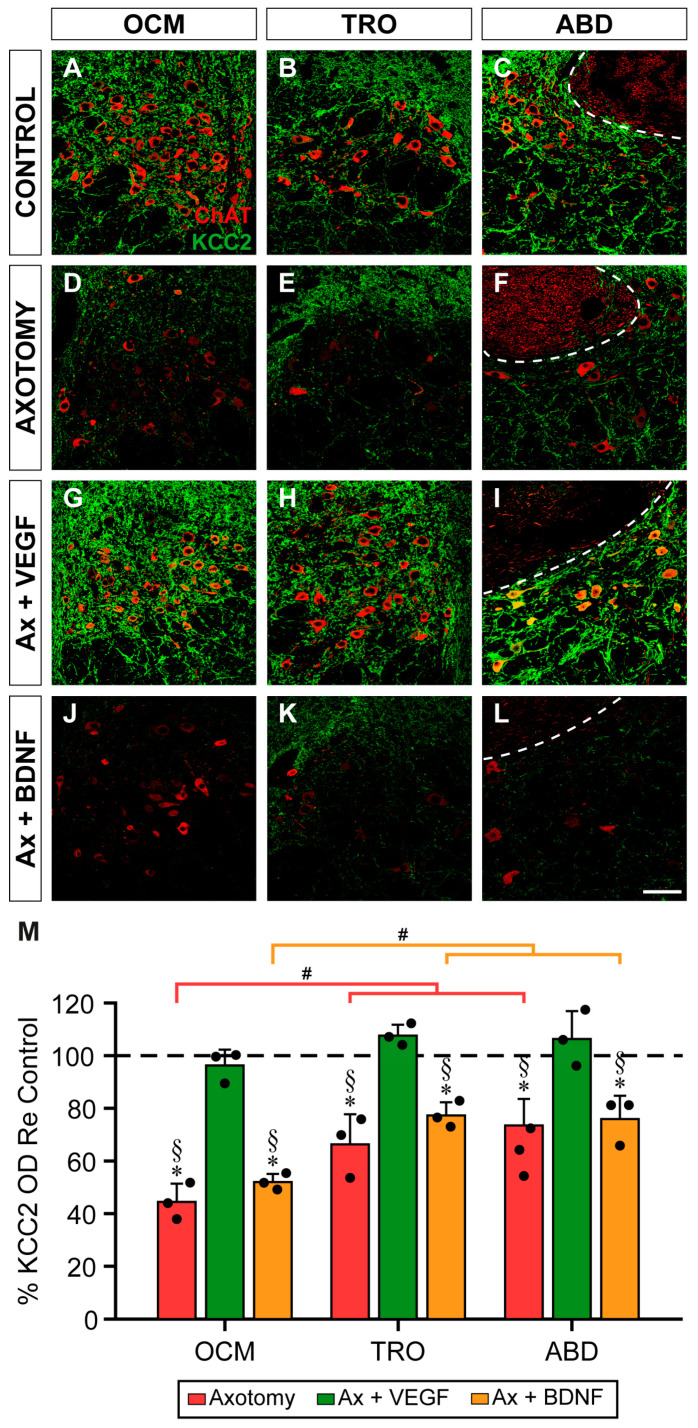
Effects of axotomy alone or together with the administration of VEGF or BDNF on the optical density of KCC2 in the neuropil of extraocular motor nuclei 15 days after axotomy. (**A**–**L**) Confocal images showing KCC2 immunostaining (green) in the neuropil of the oculomotor (OCM; (**A**,**D**,**G**,**J**)), trochlear (TRO; (**B**,**E**,**H**,**K**)), and abducens (ABD; (**C**,**F**,**I**,**L**)) nuclei, in control (**A**–**C**), and 15 days after axotomy (**D**–**F**) or axotomy (Ax) + VEGF (**G**–**I**) or BDNF (**J**–**L**). ChAT immunofluorescence (red) was used to label the motoneurons. The dashed white lines in (**C**,**F**,**I**,**L**) delimit the genu of the facial nerve. (**M**) Bar chart of KCC2 optical density (OD) measurements in the neuropil of oculomotor, trochlear, and abducens nuclei 15 days after axotomy and after axotomy + VEGF or BDNF treatment. Data are represented as percentages relative (Re) to the control side (100%, dashed horizontal line). A two-way ANOVA test followed by Fisher’s post hoc test for multiple comparisons was used to detect significant differences between groups. The following symbols were used to indicate significant differences between treatments within the same motor nucleus: *, significant difference (*p* < 0.05) compared to control; §, significant difference (*p* < 0.05) with respect to axotomy + VEGF. Hashtags were used to indicate significant differences (#, *p* < 0.05) between the three extraocular motor nuclei within the same treatment. Data correspond to mean ± SD. The number of animals per group was *n* = 3 (black dots). Scale bar is 75 µm in (**L**) for (**A**–**L**).

According to the time course of the KCC2 changes after axotomy (Figure 1), the KCC2 optical density reached its minimum at 15 days. The three motor nuclei showed similar responses to the different experimental situations by 15 days (Figure 4A–L). Thus, the neuropil of the three nuclei (Figure 4D,F) showed a remarkable descent in the intensity of KCC2 immunolabeling 15 days after injury compared to the control (Figure 4A–C). In the axotomy + VEGF situation (Figure 4G–I), the KCC2 immunofluorescence showed no change with respect to the control in the oculomotor (Figure 4A,G), trochlear (Figure 4B,H), and abducens (Figure 4C,I) nuclei. It was notorious that VEGF administration (Figure 4G–I) prevented the decay in the ChAT expression that occurs in axotomized motoneurons (Figure 4D–F). Finally, the BDNF + axotomy situation showed a decrease in KCC2 (Figure 4J–L) in all the nuclei that resembled the axotomy state (Figure 4D–F).

A two-way ANOVA test followed by Fisher´s post hoc test for multiple comparisons was carried out. Significant differences between the experimental situations (*F*_(3,24)_ = 88.406, *p* < 0.0001) and nuclei (*F*_(2,24)_ = 19.829, *p* < 0.0001) were found. Axotomy drastically reduced the KCC2 optical densities in the neuropil of the three nuclei (value states above relative to Figure 1; *p* < 0.0001) with respect to the controls (horizontal dashed line in Figure 4M).

The administration of VEGF prevented the axotomy-induced decay in KCC2 (oculomotor: 96.40 ± 6.04%, *p* = 0.5256; trochlear: 107.78 ± 4.13%, *p* = 0.1767; abducens: 106.48 ± 10.63%, *p* = 0.2577). The optical densities after VEGF treatment were also significantly higher (*p* < 0.0001) than those of axotomy in the three nuclei. By contrast, BDNF administration produced mean KCC2 optical densities (oculomotor: 52.09 ± 3.13%; trochlear: 77.44 ± 4.99%; abducens: 76.06 ± 8.86%) that were significantly lower than that of the control (*p* < 0.0001, *p* = 0.0005, *p* = 0.0003, respectively) and similar to that of the axotomy group (*p* = 0.1916, *p* = 0.0602, *p* = 0.6679, respectively). Data obtained with BDNF were also significantly lower (*p* < 0.0001 in the three groups) than those obtained with VEGF (Figure 4M).

Comparisons between the nuclei within the same experimental situation revealed that the oculomotor nucleus showed a mean KCC2 optical density after axotomy significantly lower than those obtained in the trochlear and abducens nuclei (*p* = 0.0007 and *p* < 0.0001, respectively). In turn, the KCC2 optical densities of the latter two nuclei did not differ (i.e., trochlear and abducens, *p* = 0.2092) (Figure 4M). A similar finding was obtained when comparisons between the nuclei were performed in the axotomy + BDNF situation. Thus, in the oculomotor nucleus, the decay in KCC2 was significantly greater than that obtained from the trochlear and abducens nuclei (*p* = 0.0001 and *p* = 0.0003, respectively), the latter two nuclei being similar to each other (i.e., trochlear and abducens, *p* = 0.8066) (Figure 4M).

### 2.5. KCC2 Optical Density in Motoneuron Plasma Membrane 15 Days after Axotomy and VEGF or BDNF Treatment

**Figure 5:**
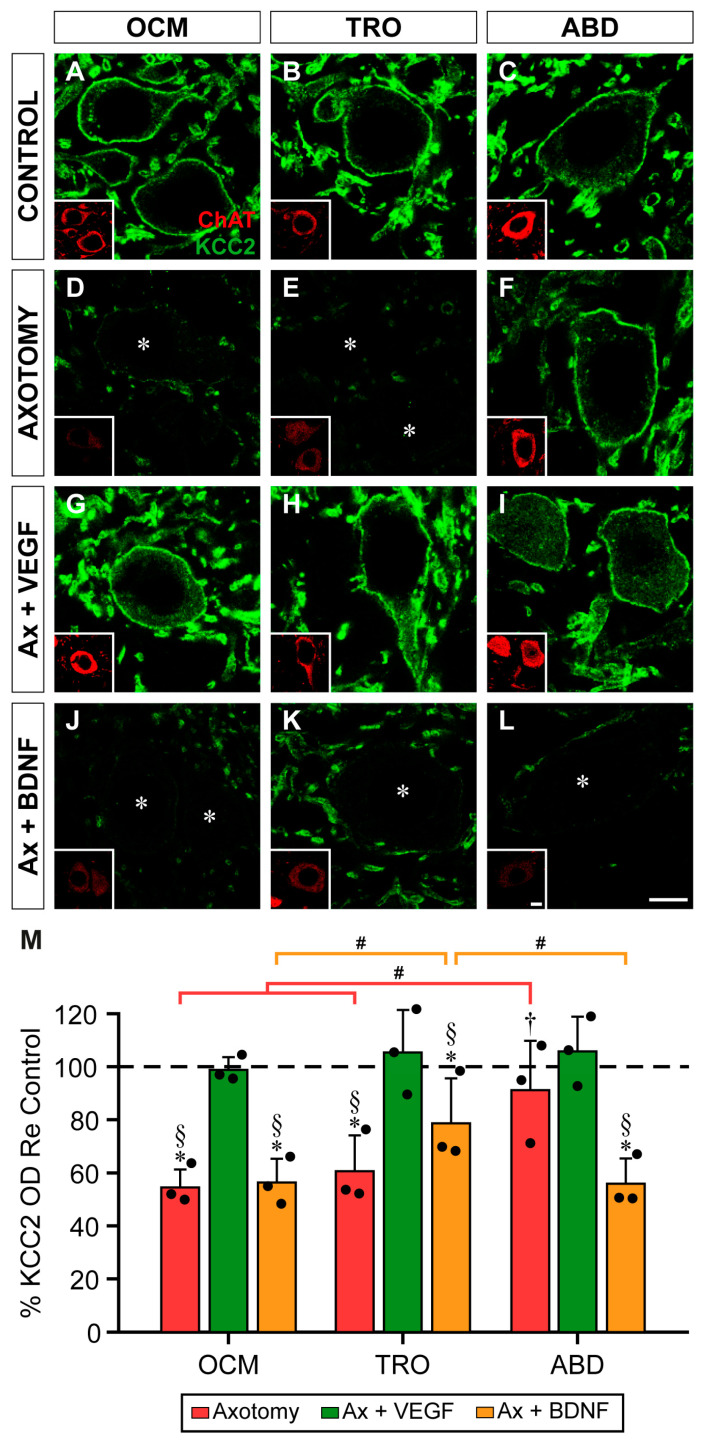
Analysis of the intensity of KCC2 immunofluorescence on the soma surface of extraocular motoneurons 15 days after axotomy and VEGF or BDNF administration. (**A**–**L**) Confocal images of oculomotor (OCM; (**A**,**D**,**G**,**J**)), trochlear (TRO; (**B**,**E**,**H**,**K**)), and abducens (ABD; (**C**,**F**,**I**,**L**)) motoneurons (identified by ChAT immunostaining, in red, inserts in each panel) showing perisomatic immunofluorescence to KCC2 (green). Images correspond to control (**A**–**C**) and 15 days after axotomy alone (**D**–**F**) or axotomy (Ax) + VEGF (**G**–**I**) or BDNF (**J**–**L**) administration. Asterisks in (**D**,**E**,**J**,**K**,**L**) indicate motoneurons with very low levels of KCC2 immunostaining around their plasma membrane. White asterisks indicate cells with low perisomatic KCC2 labeling. (**M**) Bar chart showing KCC2 optical densities (ODs) on the soma surface of oculomotor, trochlear, and abducens motoneuron cell bodies. Data are expressed as percentages relative (Re) to the control side (100%, dashed horizontal line). A two-way ANOVA test followed by Fisher´s post hoc test for multiple comparisons was used to detect significant differences between groups. To indicate significant differences between experimental situations within the same motor nucleus, we used the following symbols: *, significant difference (*p* < 0.05) compared to the control; §, significant difference (*p* < 0.05) relative to the axotomy + VEGF situation; †, significant difference (*p* < 0.05) relative to the axotomy + BDNF situation. Hashtags illustrate significant differences (#, *p* < 0.05) between the three motoneuronal types within the same experimental situation. Data correspond to mean ± SD. The number of animals per group was *n* = 3 (black dots). Scale bars are 5 µm in (**L**) for (**A**–**L**), and 5 µm in the insert in (**L**) for all inserts.

The immunofluorescence against KCC2 15 days after injury revealed that the oculomotor (Figure 5D) and trochlear (Figure 5E) motoneurons exhibited a marked decrease in staining compared to their respective controls (Figure 5A,B). As we have previously reported [32], the abducens motoneurons, strikingly, did not show a downregulation of KCC2 on their somatic surface 15 days after axotomy (Figure 5F, compared to the control in Figure 5C), when changes were more drastic (Figure 1).

In the axotomy + VEGF situation, all the motoneurons showed intense perisomatic labeling (Figure 5G–I). However, BDNF administration did not show any change compared to axotomy in the oculomotor (Figure 5J) and trochlear (Figure 5K) motoneurons. An interesting finding was that treatment with BDNF for 15 days markedly reduced the KCC2 around the cell body perimeter of the axotomized abducens motoneurons (Figure 5L).

A two-way ANOVA test was used, followed by Fisher’s post hoc test for multiple pairwise comparisons, revealing significant differences between treatments (*F*_(3,24)_ = 30.757, *p* < 0.0001), but not between nuclei (*F*_(2,24)_ = 3.181, *p* = 0.0595).

In the oculomotor motoneurons, the decrease in the perisomatic KCC2 15 days post-lesion reached a mean of 54.59 ± 6.8% (Figure 5M), a value which was significantly lower than that of the control (horizontal dashed line in Figure 5M, 100%; *p* < 0.0001). When VEGF was administered to the axotomized oculomotor motoneurons, the mean optical density of KCC2 was 98.97 ± 4.82%, which was similar to that of the control (*p* = 0.9108) and statistically higher than that of axotomy (*p* < 0.0001). By contrast, the axotomized oculomotor motoneurons treated for 15 days with BDNF showed a mean perisomatic KCC2 optical density of 56.47 ± 8.97%, a value significantly lower than that of the control (*p* < 0.0001) but similar to that of axotomy (*p* = 0.8387).

Similar findings were obtained in the trochlear motoneurons. Fifteen days after axotomy, there was a significant (*p* = 0.0002) decrease in the KCC2 optical density on the soma surface of the trochlear motoneurons (60.72 ± 13.55%) compared to the control (Figure 5M). VEGF administration to axotomized trochlear motoneurons for 15 days prevented the injury-induced downregulation of KCC2, reaching a mean optical density of 105.52 ± 16.08%, which was similar to that of the control (*p* = 0.5499) and significantly higher than that of axotomy (*p* < 0.0001). By contrast, BDNF delivery produced a mean KCC2 value of 78.80 ± 16.97%, which did not differ from that of axotomy (*p* = 0.0585) but was significantly lower than that of the control (*p* = 0.0286) and axotomy + VEGF (*p* = 0.0072) (Figure 5M). Therefore, in the oculomotor and trochlear motoneurons, VEGF prevented the axotomy-induced KCC2 downregulation, while BDNF maintained the axotomy state.

The abducens motoneurons showed a differential response to axotomy and neurotrophic factor administration. First, axotomy did not downregulate KCC2 in the somatic plasma membrane of these motoneurons at 15 days, presenting a mean optical density of 91.33 ± 18.64%, a value that was similar to that of the control (*p* = 0.3502). The administration of VEGF yielded a value (105.93 ± 13.13%) similar to those of both the control (*p* = 0.5211) and axotomy (*p* = 0.1218). The most outstanding finding in the abducens motoneurons was that the administration of BDNF to injured motoneurons decreased the KCC2 optical density on the soma surface of these motoneurons. Thus, the abducens motoneurons in the axotomy + BDNF situation showed a value of 56.0 ± 9.54%, which was significantly lower than those of the control (*p* < 0.0001), axotomy (*p* = 0.0007), and axotomy + VEGF (*p* < 0.0001) (Figure 5M).

When comparisons were performed between the nuclei within the same treatment, pairwise comparisons by Fisher’s test revealed the following differences. First, the perisomatic KCC2 optical density 15 days after the lesion was significantly higher in the abducens motoneurons than in the oculomotor (*p* = 0.0005) and trochlear (*p* = 0.0026) motoneurons. Second, in the axotomy + BDNF situation, the value obtained in the trochlear motoneurons was significantly higher than those obtained in the oculomotor (*p* = 0.0218) and abducens (*p* = 0.0194) motoneurons (Figure 5M).

## 3. Discussion

**Figure 6:**
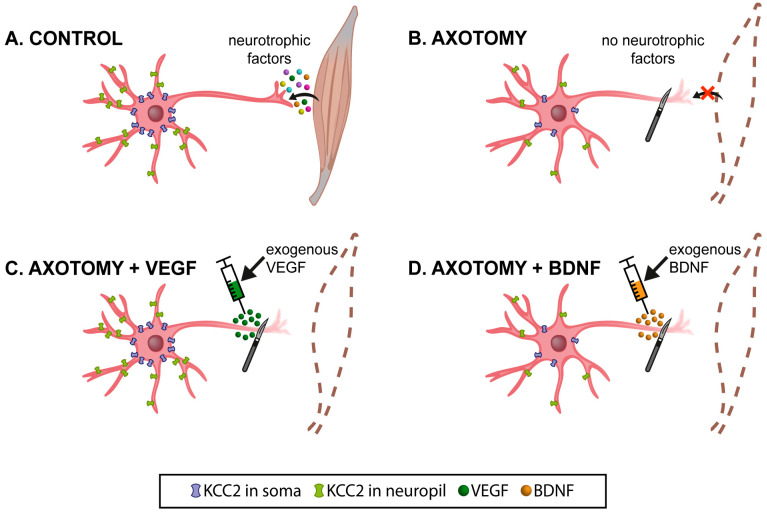
Schematic illustration of the present results at 15 days post-lesion (when KCC2 levels decreased to a minimum). (**A**) Control extraocular motoneurons (oculomotor, trochlear, and abducens) contained the cotransporter KCC2 in the plasma membrane of their dendrites (neuropil; in green) and cell bodies (in blue). These motoneurons receive neurotrophic factors retrogradely from their target muscle. (**B**) After axotomy, motoneurons were deprived of neurotrophic retrograde supply (red x). Muscles were also removed (dashed brown lines). Axotomy induced a remarkable decay in the levels of KCC2 in the neuropil and on soma surface of motoneurons. The only exception was the abducens motoneurons at the somatic surface level. (**C**) VEGF administration to axotomized motoneurons prevented the KCC2 decrease induced by lesion, at the level of the neuropil and soma surface. (**D**) By contrast, BDNF administration to axotomized motoneurons did not modify the injury-induced low KCC2 levels observed in the neuropil and on the soma surface. However, it should be emphasized that, in the particular case of the somatic membrane of abducens motoneurons, whose KCC2 levels did not change after injury, BDNF caused a significant downregulation of this cotransporter. Taken together, our results suggest that VEGF is an important neurotrophic factor involved in maintaining adequate levels of KCC2 along the somatodendritic membrane of these motoneurons, while BDNF might play a relevant role mediating the downregulation of KCC2 after injury.

The present results show that axotomy induced a general decrease in the KCC2 levels in the extraocular motoneurons, both at the soma surface and in the neuropil (dendritic compartment). This was particularly evident at 15 days after injury. The most remarkable finding of the present work was the ability of VEGF to prevent the decrease in KCC2 induced by injury. In contrast, BDNF administration maintained the low levels of KCC2 induced by axotomy or further reduced KCC2 below the axotomy levels (i.e., oculomotor neuropil, 7 days) or, in those cases in which axotomy did not downregulate KCC2, then BDNF did induce a significant KCC2 decline (i.e., abducens soma, 15 days). The fact that BDNF clearly induced the downregulation of KCC2 in some particular groups of the present study opens up the possibility that this factor might be mediating the decrease in KCC2 mostly observed in axotomized extraocular motoneurons. However, this hypothesis requires further investigation to be contrasted. In addition, the present work shows a noticeable difference in the effects obtained after the administration of VEGF or BDNF. Whereas VEGF prevented KCC2 downregulation, BDNF either did not change or induced a reduction in the levels of KCC2 in the axotomized extraocular motoneurons (Figure 6).

### 3.1. Time Course of Changes in KCC2 after Axotomy

The present results have shown that KCC2 is transiently downregulated after the axotomy of extraocular motoneurons, as the KCC2 levels recover to normal over time. In detail, in all three extraoculomotor nuclei, the KCC2 optical density on the soma surface of the motoneurons decreased to a minimum at 15 days post-injury. A tendency towards recovery was subsequently observed at 28 days, but complete recovery occurred at 60 days, both in the neuropil and in the plasma membrane of the motoneurons and for the three nuclei: oculomotor, trochlear, and abducens. Similarly, ChAT expression in axotomized extraocular motoneurons also decays and spontaneously recovers 4 weeks after injury, as we have previously reported [46].

Other cranial (i.e., facial, hypoglossal) and spinal motoneurons also experience a downregulation of their KCC2 levels after axotomy, reaching a minimum expression between 14 and 21 days after injury, and a spontaneous recovery around 60 days [25,26,29,30], in accordance with our present results. Interestingly, the recovery of the KCC2 content in facial, hypoglossal, and spinal axotomized motoneurons occurs in parallel with axonal regeneration and muscle reinnervation [25,26,29,30]. In fact, when axonal regeneration is not allowed and muscle reinnervation is prevented, then there is no recovery of the KCC2 levels, as reported for spinal motoneurons after sciatic nerve ligation, so that 60 days after injury, the KCC2 contents in axotomized and ligated motoneurons show values that are significantly lower than those of the control [25]. Altogether, these observations suggest that muscle-derived neurotrophic factors might play an important role in the recovery of KCC2 after motoneuron injury. Along this line, the present data show that the administration of VEGF to axotomized extraocular motoneurons (delivered retrogradely from the orbit) prevented the downregulation of KCC2 (see below).

If target-derived neurotrophic factors are indeed relevant molecules for the maintenance of adequate KCC2 levels, it could be argued that the spontaneous re-establishment of normal KCC2 levels in the axotomized extraocular motoneurons observed 60 days after injury could reflect the reinnervation of new target cells. Since the axotomy procedure used in the present work also resulted in the removal of eye muscles, muscle reinnervation was prevented, but the sectioned axons might have reinnervated nearby orbital tissues. Alternatively, neurotrophic factors could originate from different sources. As we have discussed extensively in our previous paper [46] in relation to ChAT recovery in axotomized extraocular motoneurons, trophic molecules could arise from different sources in addition to target cells. Thus, glial cells from the periphery (Schwann cells) or in the CNS (e.g., astrocytes, microglia) could supply trophic factors to the axon or cell body of severed motoneurons, using the paracrine pathway. Afferent inputs may also release trophic factors (anterograde pathway) and, finally, these molecules could be delivered in an autocrine manner. Although the recovery of KCC2 in axotomized facial, hypoglossal, and spinal motoneurons seems to be due to muscle reinnervation [25,26,29,30], KCC2 restoration in extraocular motoneurons is likely produced by neurotrophic molecules whose origin remains unknown.

The present findings have demonstrated a clear difference in the KCC2 changes over time after axotomy between the abducens and other two extraocular motor nuclei. In particular, the KCC2 content in the plasma membrane of the abducens motoneurons did not change at any time point studied after injury (7, 15, 28, and 60 days), in marked contrast to the oculomotor and trochlear motoneurons, which showed a strong and significant reduction at 15 days, consistent with our previous work conducted on cats and rats [32]. The preservation of KCC2 in abducens motoneurons after axotomy could be related to their different embryological origin compared to oculomotor and trochlear motoneurons [47], or to different neurotrophic requirements or other sources of neurotrophic factors.

### 3.2. VEGF Prevented KCC2 Downregulation in Axotomized Extraocular Motoneurons

The most outstanding result of the present work was that VEGF administration prevented the KCC2 decrease induced by axotomy in the extraocular motoneurons. This effect occurred in both the neuropil and soma, and it was more evident at 15 days than at 7 days. However, unlike the oculomotor and trochlear motoneurons, the abducens motoneurons did not show axotomy-induced KCC2 depletion, as discussed above.

It is interesting to note that, in a cat, VEGF upregulated the perisomatic KCC2 in axotomized abducens motoneurons above the control values [32]. We have also shown that this KCC2 upregulation is accompanied by an increase in the inhibitory eye-related signals in the discharge of abducens motoneurons recorded under alert conditions [32]. These physiological data are in agreement with a larger chloride extrusion and therefore an enhancement of synaptic inhibitory inputs. In the present results, the axotomized abducens motoneurons treated with VEGF also showed a small increase above the control levels at 15 days, but this did not reach significance. This might suggest a differential response of rat versus cat abducens motoneurons to VEGF. In consequence, VEGF in the rat likely restored the normal excitatory/inhibitory synaptic balance in abducens and also other injured extraocular motoneurons.

We have also previously demonstrated that VEGF is an essential neurotrophic factor for extraocular motoneurons. The alterations in the firing and synaptic properties induced by axotomy in cat abducens motoneurons are fully restored following the exogenous administration of VEGF [48]. Moreover, the retrograde intramuscular delivery of a neutralizing VEGF antibody to intact, uninjured abducens motoneurons produces severe changes in their discharge characteristics and synaptic inputs that resemble the axotomy situation [49]. In this context, it is interesting to consider that the deficit of VEGF in mutant mice (VEGF^δ/δ^) led to motor weakness and muscle atrophy produced by the degeneration of spinal motoneurons in a model that resembles amyotrophic lateral sclerosis [50]. All these data point to VEGF as a crucial neurotrophic factor for motoneurons [51]. Future research is needed to elucidate whether VEGF delivery could also rescue the injury-induced downregulation of KCC2 in spinal motoneurons.

The mechanism by which VEGF exerts its neuroprotective action has been described previously [52] in spinal motoneurons exposed to excitotoxicity. VEGF acting on its receptor VEGFR-2 activates the pathways of PI3-K/Akt and MEK/ERK [53]. Interestingly, these signaling mechanisms are shared by BDNF/TrkB to upregulate KCC2 (see below) [37,43].

### 3.3. Effects of BDNF Administration on KCC2 Levels of Axotomized Extraocular Motoneurons

The administration of BDNF to axotomized motoneurons produced diverse responses depending on the motoneuronal type and time post-lesion. Overall (particularly at 15 days after injury), it was observed that, in those cases in which KCC2 was downregulated by axotomy, the supply of BDNF did not produce any effect; that is, BDNF maintained the low KCC2 levels induced by axotomy (except in the oculomotor neuropil at 7 days post-lesion where BDNF delivery further reduced the KCC2 levels). By contrast, when KCC2 was not modified by axotomy (perisomatic KCC2 in oculomotor motoneurons at 7 days and in abducens motoneurons at 7 and 15 days), the response to BDNF administration was remarkably a reduction in the KCC2 levels. These data might suggest that BDNF participated in the post-injury downregulation of KCC2 in the extraocular motoneurons, in agreement with previous literature. A large body of evidence has previously shown that BDNF downregulates KCC2 in different neuronal types and lesion models [42,44]. For instance, hippocampal neurons exposed to seizures induced by kindling in vivo, or the addition of BDNF in vitro from intact tissue, experience a downregulation in KCC2 mRNA and protein that is mediated by TrkB signaling [43,54]. In a mouse model of viral-induced epilepsy, the BDNF levels in the hippocampus increased, which led to a decrease in KCC2 followed by hyperexcitability and seizures [55]. BDNF-TrkB signaling is also involved in the downregulation of KCC2 in epileptic patients [17]. Similarly, spinal dorsal horn neurons experience a decrease in KCC2 after peripheral nerve injury or the injection of formalin (leading to hyperalgesia) that is mediated by BDNF. The administration of inhibitors of BDNF or its signaling receptor TrkB prevents KCC2 downregulation and reduces pain [39,40,41,45]. Even the administration of BDNF intrathecally in uninjured animals provokes allodynia and decreases KCC2 expression [41]. In this context, it is worth mentioning that in some neurological diseases, such as epilepsy, neuropathic pain, and spinal cord injury, a downregulation in KCC2 has also been shown to be mediated by BDNF, which underlies the characteristic hyperexcitability of these diseases due to the depolarizing activity of GABA [17,18,42,43,54]. Accordingly, selective antagonists of TrkB receptors have been suggested as likely therapeutic agents for the treatment of these neurological disorders [14,15,16,56].

However, previous reports have also shown opposite results indicating that BDNF upregulates KCC2 in lesion models such as epilepsy [36], spinal cord injury [37,57], and axotomized corticospinal neurons [35]. Some authors have postulated that BDNF decreases KCC2 in intact adult neurons, while it increases KCC2 in injured adult neurons, as occurs during development [33,58], considering that the neurons return to an “immature” or “dedifferentiated” state [21,37,59].

Although facial [28,29], hypoglossal [30], vagal [31], and spinal [25,27] motoneurons experience a downregulation in KCC2 after axotomy, the regulation of KCC2 by BDNF has been evaluated only in spinal motoneurons. It has been demonstrated that KCC2 downregulation in axotomized spinal motoneurons is not altered by the deletion of *bdnf* specifically from microglia or motoneurons, or the pharmaco-genetic block of TrkB [25]. However, only one time point coincident with maximal KCC2 depletion was examined. Our results suggest that exogenous BDNF accelerates this process, maybe by promoting KCC2 removal from the membrane in the context of a lack of gene expression.

The fact that BDNF can induce either the downregulation or upregulation of KCC2 under different situations likely depends on the particular signaling pathway activated. BDNF-TrkB downstream cascades involve both Shc/FRS-2 and PLCγ. The downregulation of KCC2 occurs when the Shc pathway is activated and PLCγ is present. The upregulation of KCC2 is triggered by the Shc pathway when PLCγ is absent [37,43,44,59]. The exact pathways triggered in different types of axotomized motoneurons are currently unknown.

In conclusion, we found that VEGF or BDNF administration acts differentially on axotomized extraocular motoneurons. VEGF administration prevented the decrease in KCC2 induced by axotomy. By contrast, BDNF supply, in general, maintained or reduced the low KCC2 levels induced by axotomy. Moreover, BDNF downregulated KCC2 whenever axotomy did not reduce the levels of this cotransporter (e.g., abducens motoneuron cell bodies, 15 days post-lesion). The present results indicating that VEGF and BDNF modify the KCC2 levels in axotomized motoneurons raise an intriguing question about the possible effects of these two neurotrophic factors on the expression of the cotransporter that mediates the opposite chloride flow in neurons (i.e., NKCC1). This question requires further research to be resolved. Finally, the finding that VEGF prevented an injury-induced decrease in KCC2 has considerable clinical relevance, as it could act as a potential therapeutic agent for the treatment of neurological diseases characterized by the downregulation of KCC2.

## 4. Materials and Methods

Adult Wistar rats were obtained from the animal house of University of Sevilla. All experimental procedures were performed following the guidelines of the European Union (2010/63/EU) and the Spanish legislation (R.D. 53/2013, BOE 34/11370-421) and were ethically approved (04/07/2022/098) for the refinement of the procedures and a reduction in the number of animals.

### 4.1. Animals and Surgical Procedures

A total of *n* = 24 female Wistar rats (2–3 months old) were used in the present work. The experimental groups included lesioned animals treated with vehicle and sacrificed at 7, 15, 28, or 60 days (axotomy alone; 12 animals), and lesioned animals treated with either VEGF or BDNF sacrificed at 7 or 15 days (axotomy + treatment; 12 animals). The number of animals used in each group was *n* = 3. Surgery was performed under deep anesthesia (ketamine, 80 mg/kg, and xylazine, 10 mg/kg, i.p.) and consisted in the enucleation of the left eye to induce the axotomy of the motoneurons innervating the extraocular muscles on that side. The procedure for enucleation has been described in detail previously [46,60,61,62]. Briefly, the left eyeball was surgically excised, including all extraocular muscles. The orbit was then filled with an absorbable gelatin sponge pellet (Gelfoam, Pfizer, Belgium) soaked in vehicle [(20 µL of phosphate-buffered saline (PBS) at pH 7.4, containing 0.1% bovine serum albumin (BSA)] or VEGF (1 µg of VEGF dissolved in 20 µL of PBS with 0.1% BSA) or BDNF (1 µg of BDNF dissolved in 20 µL of PBS with 0.1% BSA). These doses were selected according to our previous findings in electrophysiological experiments [48,63]. The recombinant rat VEGF_164_ amino acid form or the recombinant human BDNF (both from R&D Systems, Minneapolis, MN, USA) were applied to injured animals, which were allowed to survive for 7 or 15 days. The groups that prepared for a survival of 15 days received a second dose of factors by 7 days. A time course of KCC2 changes was studied at 7, 15, 28, and 60 days post-lesion. These animals received the same surgery as described above but received only vehicle. After the implant, eyelids were sutured. Animals received postoperative care daily and were treated with anti-inflammatories and analgesics (meloxicam, 2 mg/kg, s.c.) and antibiotics (amoxicillin, 10 mg/kg, i.m.) as needed.

At the scheduled end-points, the animals were sacrificed by an overdose of sodium pentobarbital (100 mg/kg, i.p.) and the vascular system was perfused transcardially with 0.9% saline followed by 4% paraformaldehyde in PB. The brainstem was extracted, postfixed for 2 h, and cryoprotected by immersion in 30% sucrose solution in PBS. The brainstem was then cut into 30 µm thick coronal sections on a cryostat (Leica CM 1850, Wetzlar, Germany).

Extraocular motoneurons are located in three brainstem nuclei: the oculomotor (III), trochlear (IV), and abducens (VI) nuclei. The oculomotor nucleus contains four motoneuronal pools: the motoneurons innervating the inferior rectus, the medial rectus, and the inferior oblique muscles, which project ipsilaterally, and the superior rectus subdivision, which innervates the muscle contralaterally. Since superior rectus motoneurons are located caudally in the rat oculomotor complex, we selected sections from the rostral half of the nucleus. The trochlear nucleus innervates the superior oblique muscle contralaterally, and the abducens nucleus contains the motoneurons innervating the lateral rectus muscle ipsilaterally [64].

### 4.2. Immunofluorescence and Quantification

Free-floating brainstem sections were processed sequentially for double immunofluorescence. The primary antibodies used were goat polyclonal anti-ChAT, a marker for motoneurons (1:200; Merck-Millipore, Darmstadt, Germany), and rabbit polyclonal antibody against the rat KCC2 cotransporter (1:250; Millipore) in a region shared by KCC2a and KCC2b isoforms [65,66]; thus, the antibody recognized both isoforms. Secondary antibodies were obtained from Jackson ImmunoResearch (1:50; West Baltimore Pike, West Grove, PA, USA) and were the following: (i) donkey anti-goat IgG coupled to TRITC (for ChAT detection), and (ii) donkey anti-rabbit IgG coupled to FITC (for KCC2 detection).

After immunofluorescence, sections were visualized using a confocal laser scanning microscope (Zeiss LSM 7 DUO; Oberkochen, Germany). Images were captured at the level of the three extraocular motor nuclei using X40 and X63 objectives, in order to analyze the KCC2 optical density at the neuropil level (area devoid of cells, mostly the dendritic compartment) and somatic level, respectively. Acquisition parameters were adjusted to maximize the dynamic range of the images. Series of focal planes of a 0.75 µm virtual thickness were captured through the regions of interest. Z-stacks were generated by overlapping 4 focal planes of the same histological section using the imaging software ZEN 3.8 (Zeiss; Overkochem, Germany). Confocal images were analyzed using the ImageJ program (https://imagej.net/ij/, NIH, Bethesda, MD, USA).

For the quantification of KCC2, we used between 3 and 4 histological sections per animal and per extraoculomotor nuclei (i.e., 9–15 total sections per animal). To quantify the intensity of the KCC2 immunofluorescence on the motoneuron surface (i.e., perisomatic optical density), focal planes were selected at the mid-nuclear level. In each section, background measurements for soma KCC2 quantification were taken from 4 regions of interest (ROIs) of 25 µm^2^ in the neuropil in the same optical plane, next to the motoneurons and lacking any somatic or dendritic labeling. The average KCC2 optical density for each cell was measured on the perimeter of the motoneuronal somata after background correction. KCC2 immunofluorescence was measured on both the control and the operated sides. Subsequently, the KCC2 optical density on the somatic surface of the lesioned motoneurons was normalized to that of the control motoneurons from the same histological section and expressed as a percentage. The number of motoneurons analyzed per animal varied between 135 and 252, with a mean of 203.22 ± 35.41 (SD).

To analyze the optical density in the neuropil, the intensity of the KCC2 immunofluorescence was measured in 20 ROIs of 20 × 20 µm on each side of the histological section and in each extraocular motor nucleus, identified by the presence of ChAT-immunoreactive motoneurons, but avoiding cell profiles. Background correction was carried out measuring neuropil areas devoid of labeled cell profiles for each image (4 ROIs per side). Thereafter, data were normalized to the control side of the same section and expressed as percentages. The number of ROIs analyzed per animal varied between 360 and 480, with a mean of 391.11 ± 40.13 (SD).

### 4.3. Statistics

Statistical comparisons were performed by one-way or two-way ANOVA tests using the program GraphPad Prism 10.1.2 (GraphPad Software, Dotmatics, Boston, MA, USA), at an overall significance level of *p* < 0.05. Data were expressed as mean ± SD.

## Data Availability

The data presented in this study are available from the corresponding author upon reasonable request.
